# Metabolic manipulation in Dilated Cardiomyopathy: assessing the role of trimetazidine

**DOI:** 10.1186/1749-8090-10-S1-A7

**Published:** 2015-12-16

**Authors:** Aditya Kapoor, Suman Jatain, Surendra K Agarwal, Shantanu Pande, Archana Sinha, Rooplai Khanna, Sudeep Kumar, Naveen Garg, Satyendra Tewari, Pravin Goel

**Affiliations:** 1 Department of Cardiology, Sanjay Gandhi PGIMS, Lucknow 226014, India; 2 Department of Cardiovascular-Thoracic Surgery, Sanjay Gandhi PGIMS, Lucknow 226014, India

## Background/Introduction

Altered substrate metabolism plays an important role in pathophysiology of heart failure (HF). Optimization of myocardial energy metabolism with metabolic modulators like trimetazidine (TMZ) allows more efficacious energy production.

## Aims/Objectives

Although TMZ has been studied extensively in patients with ischemic HF, more data are needed on its role in dilated cardiomyopathy (DCM).

## Method

100 patients of DCM (mean age 47.7 yrs, NYHA class 2.17, LVEF 27.3%) were randomized to TMZ (20 mg tid, n = 50) vs conventional therapy (n = 50). Functional status, BNP & echocardiographic parameters were assessed at 3-6 months.

## Results

Baseline characteristics were comparable among the two groups. At three months, patients on TMZ had significant improvement in mean NYHA class (2.25 vs 1.85, p = .001), 6 min walk test (349.7vs 402 m, p = 0.001), LVD-36 score (25.5 vs 21, p = .001) and fall in BNP (744.7 vs 248.3 pg/ml, p = .001). This was accompanied by significant improvement in indexed LV end-systolic (LVESV, 87.1 ± 27.5 vs 78.5 ± 24.9 ml/m2, p = 0.0001) and LV end-diastolic volumes (LVEDV, 117.6 ± 29.3 vs 110.9 ± 27.4 ml/m2, p = 0.0001) and LVEF (27vs30.9%, p = .0001) along with reduction in LV wall stress (90.2 ± 18.9 vs 71.1 ± 13.2 dyn/cm2, p = 0.0001). Other echocardiographic parameters also improved after three 3 month of TMZ (E/A ratio, E/A VTI, Myocardial performance index) and TDI parameters (E/e'septal, and E/e' lateral). Patients not on TMZ had no significant change in NYHA Class, LVD-36 scores, LV volumes or LVEF at 3 months although BNP levels & LV wall stress reduced, albeit to a lesser extent than TMZ. Patients on TMZ had further improvement in NYHA Class, 6 min walk test, BNP levels & all echocardiographic parameters at 6 months.

## Discussion/Conclusion

Metabolic modulators like trimetazidine have a potential role to play in altering LV remodelling and improving LV function in DCM. In this study, benefit was noted by 3 months with further improvement at 6 months.

